# Non-Redfieldian dynamics driven by phytoplankton phosphate frugality explain nutrient and chlorophyll patterns in model simulations for the Mediterranean Sea

**DOI:** 10.1016/j.pocean.2019.02.005

**Published:** 2019-04

**Authors:** Diego Macias, I. Emma Huertas, Elisa Garcia-Gorriz, Adolf Stips

**Affiliations:** a European Commission, Joint Research Centre, Via E. Fermi, Ispra, Varese, Italy; b CSIC, Instituto de Ciencias de Andalucía, Avd. Republica Saharaui, Puerto Real, Cádiz, Spain

**Keywords:** Mediterranean Sea, Biogeochemical modeling, Non-Redfieldian dynamics, Nutrients plasticity

## Abstract

The relative abundance of nitrate (N) over phosphate (P) measured as a molar ratio (N:P) is typically considered to determine the macronutrient limiting marine primary production. In low-complexity biogeochemical models, a simple threshold value is usually applied based on the canonical Redfield ratio (N:P = 16). However, the N:P ratio is not constant in many oceanic areas, especially marginal, semi-enclosed seas, such as the Mediterranean basin. In this work, a flexible definition of the N:P ratio based on the capacity of phytoplankton to modulate phosphate uptake according to its availability in seawater, the so-called *Line of Frugality*, is incorporated into the biogeochemical model MedERGOM. This modification allows the acquisition of a more realistic representation of the stoichiometry of nutrients in the Mediterranean basin and allows to better reproduce the observed phytoplankton biomass in productive areas such as the Gulf of Gabes and the Adriatic Sea. This approach is, thus, especially suitable for coastal areas in which basin-scale biogeochemical models fail to reproduce patterns observed by remote sensing or in situ measurements. Our results show that implementation of the stoichiometric flexibility of phytoplankton in a low-complexity biogeochemical model enhances the reproducibility of ecosystem dynamics without increasing the computational demand, representing a simple approximation easily implemented in models aiming to describe regions with a Non-Redfieldian stoichiometry.

## Highlights

• Fixed nutrient ratio not appropriate to simulate the chemical conditions in the Mediterranean Sea.
• A simple approach allows to apply the ‘line of frugality’ to biogeochemical models.
• Nutrient and plankton simulations improve with the variable internal nutrient ratio.

## 1 Introduction

Two major ‘macronutrients’ typically limit and control biological production in marine ecosystems, nitrate (N) and phosphate (P). Both are essential components of living organisms, such as amino acids and proteins (N) or nucleic acids (P), and their relative molar ratio (N:P) is typically used to assess the limiting element for primary production in ocean regions (Smith, 1984).

A quasi-constant value of the N:P ratio in both seawater (N:P_water_, i.e., inorganic nitrate to phosphate ratio in the seawater) and marine organic matter (N:P_OM_, i.e., organic particulate nitrate and phosphate) was first established by Redfield (1934) and set to be equivalent to ∼16. The rationale for this specific value was not fully stated in Redfield’s seminal work as the ratio was only proposed to be related to ‘*the characteristics of protoplasm in general*’ (Redfield, 1934). Later, works have indicated that the N:P_OM_ value is determined by the interdependence of two universal life processes: the syntheses of rRNA and of proteins (*e.g.*, Falkowski, 2000, Geider and La Roche, 2002, Sterner and Elser, 2002, Elser et al., 2000, Klausmeier et al., 2004). Thus, the internal physiology of marine phytoplankton tends to set the N:P_OM_ at a value close to 16, which in turn influences the uptake rate of nutrients and hence their free concentration in seawater (ultimately resulting in the equivalent N:P_water_). Another alternative explanation for the Redfield ratio was developed by Tyrell (1999), who indicated that it could be created by the balance of energy-consuming N-fixation and denitrification. In ecosystems with N:P_water_ below Redfield, N-fixing organisms outcompete ordinary phytoplankton, dragging N:P_water_ upwards until the phytoplankton can thrive. The produced phytoplankton biomass will subsequently be decomposed, reducing oxygen levels and allowing denitrification to occur, dragging N:P_water_ downwards and allowing N-fixing organism to bloom and re-initiate the cycle.

Henceforth, to identify the nutrients responsible for the limitation of primary production, the ambient concentrations of nitrogen and phosphorus in seawater are typically compared with the fixed Redfield ratio of 16; if the N:P_water_ exceeds Redfield, phosphate is considered to be constraining production, and conversely, if N:P_water_ remains below Redfield, production is restricted by nitrogen. This simple approach based on a unique threshold value is usually used to reproduce the ecosystem dynamics in low complexity biogeochemical models (such as those used in global-scale applications), as it saves computational time by avoiding simulations of the metabolic routes of each nutrient separately. Loladze and Elser (2011) concluded that ‘*under optimal conditions and based on the best available data for key model parameters, this ratio also quantitatively corresponds to the canonical N:P value of 16*′.

However, there is growing evidence that both N:P_water_ and N:P_OM_ vary regionally throughout the world’s ocean (Geider and La Roche, 2002, Karpinets et al., 2006, Fransner et al., 2018), especially in marginal seas in which nutrient scarcity and/or an unbalanced supply from external sources create very particular chemical environments (Fransner et al., 2018). In these areas, biogeochemical models must consider shifts between phosphorous and nitrogen limitations, changes in nutrient loads (from ultraoligotrophic to eutrophic waters) and marked physico-chemical gradients, such as those occurring in the vicinity of river plumes (Baretta et al., 1995, Bauer et al., 2013, Blackford et al., 2004).

This is also the case of the Mediterranean Sea, where a N:P_water_ well above Redfield has been typically reported (Tanaka et al., 2011). The cause of this unusually high N:P_water_ in the Mediterranean Sea is not yet fully understood, and two contrasting hypotheses have been proposed to account for the observed trends. Early explanations refer to relatively high nitrogen fixation rates in the basin due to the activity of seagrasses and N-fixing phytoplankton (*e.g.,* Béthoux et al., 1992, Béthoux et al., 1998, Béthoux et al., 2002, Sarmiento et al., 1988, Gruber and Sarmiento, 1997, Pantoja et al., 2002, Ribera d’Alcalá et al., 2003), although later works found rather low values for pelagic N-fixation in field measurements of the Mediterranean Sea (Krom et al., 2010, Powley et al., 2017). More recent studies point to the unbalanced nutrient ratio (N:P > 16) characterizing the major external nutrient sources for the Mediterranean, such as atmospheric deposition or river discharge (Krom et al., 2004, Krom et al., 2010, Huertas et al., 2012) and to the very low denitrification rates in the Eastern Mediterranean (Krom et al., 2010, Powley et al., 2017) as the most likely explanations for the special nutrient conditions in this ultra-oligotrophic basin.

Regardless of the reasons for the nutrient ratio deviation, it seems clear that the use of a fixed N:P_OM_ = 16 for modeling biogeochemistry in such areas is not an appropriate assumption (*e.g.*, Flynn, 2010). Biogeochemical models typically deal with the plasticity of the nutrient ratio by simulating individual metabolic routes for each incorporated nutrient independently. This approach is used by high-complexity, plankton-functional-types (PFT) models, such as ERSEM (Baretta et al., 1995) and BFM (Vichi et al., 2007a, Vichi et al., 2007b). However, this methodology greatly increases the number of parameters in the model (which are difficult to constrain), as well as the computational time for model integration, making PFT models cumbersome for large areas and 3D ocean circulation applications, with some notable exceptions (*e.g.,* Ayata et al., 2014, Baretta et al., 1995, Tagliabue and Arrigo, 2005, Vichi et al., 2007a).

Galbraith and Martiny (2015) recently presented an analysis of the global relationship between ambient nutrient concentrations in seawater and the value of N:P_OM_. Briefly, these authors found that the relative ratios of nitrate to carbon (N:C) and phosphate to carbon (P:C) in the marine organic matter were dependent on the individual nutrient concentrations (N or P) in the water. The N:C vs N relationship was quite stable, changing only slightly at very low N concentrations. The P:C vs P function was, however, very dynamic across the entire range of typical P concentrations in the ocean. According to Galbraith and Martiny (2015), as P levels in water increase, the amount of P per C unit found in marine organic matter also rises, following a linear relationship that was coined the ‘*Line of Frugality’* (LoF). This global pattern reflects the higher phytoplankton plasticity for N in relation to P (DeVries and Deutsch, 2014), which can be related to the different organic compounds in which both nutrients are involved (Geider and La Roche, 2002), the energetic costs associated with the uptake and assimilation of nitrate under ammonium limitation, the ammonium toxicity (Glibert et al., 2016) or the P storage capability as a luxury type of uptake by some plankton species (*e.g.,* Klausmeier et al., 2008).

Combining the N:C vs N and the P:C vs P values, Galbraith and Martiny (2015) created a global map of N:P_OM_ based on reported values of N and P concentrations in surface waters. The N:P_OM_ distribution map showed that large deviations from 16:1 (i.e., Redfield) are expected in many oceanic regions, such as the central anticyclonic gyres and marginal seas (where the ratio is >16) and around polar areas (both Arctic and Antarctic, where the ratio is <16). The previously well-known characteristic stoichiometry of the Mediterranean Sea with a N:P_OM_ ratio well above the Redfield value (*e.g.,* Tanaka et al., 2011) was also evidenced in the distribution of Galbraith and Martiny (2015). This biogeochemical feature also occurs in other oligotrophic regions in which phytoplankton rely on N-rich proteins to gather scarce resources given the relative scarcity of phosphorous (Sterner and Elser, 2002).

Therefore, in accordance with Galbraith and Martiny (2015) and other evidence (Tanaka et al., 2011, Richon et al., 2018), the assumption of a fixed Redfield in models aimed at reproducing biogeochemistry in the Mediterranean basin does not seem to be an optimal option to simulate the observed dynamics. Instead, the assumption of the occurrence of a LoF (Galbraith and Martiny, 2015) opens up a new possibility as it allows the use of simpler and low-complexity biogeochemical models that are compatible with the existence of phytoplankton plasticity for nutrient uptake and internal quotas.

In this work, the LoF concept was introduced for the first time into a biogeochemical model that was specifically developed for the Mediterranean Sea, the so called MedERGOM model (Macías et al., 2014a), to assess its suitability to account for the variable N:P_OM_ ratio reported in the basin. The outputs of the MedERGOM simulations obtained with a constant Redfield ratio and with a variable LoF were compared for a common data-rich 8-year period (2001–2007). This approach is also suitable to explore how changes in chemical conditions (nutrient content) in the basin influence phytoplankton biomass levels, both in the horizontal and vertical dimensions, which may have implications in light of the biogeochemical alterations predicted in the Mediterranean Sea (*e.g.*, Macías et al., 2015) under climate change scenarios (IPCC 2013).

## 2 Materials and methods

### 2.1 The marine modeling framework

**Fig. 1:**
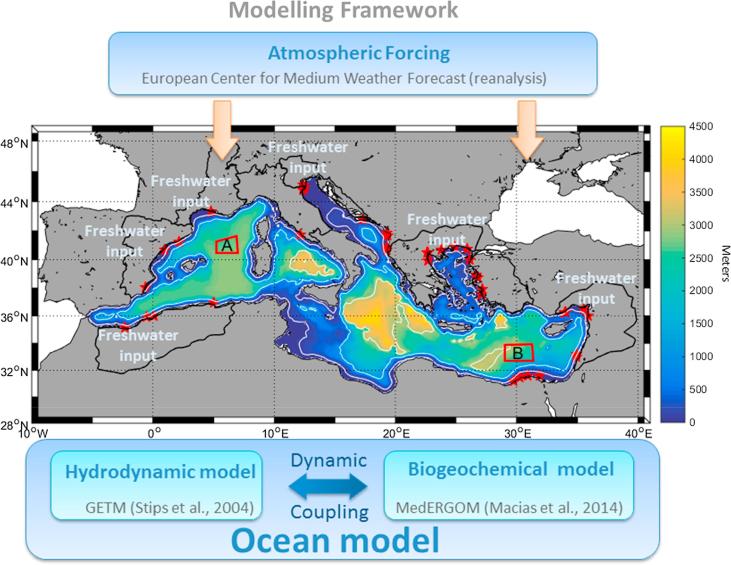
Schematic representation of the Marine Modelling Framework in its implementation to the Mediterranean Sea. Color background and white lines represent bathymetry, black lines on land represent the included rivers’ districts, red stars mark the position of the rivers mouths and red poligons (‘A’ and ‘B’) are the two open-sea selected sites for showing vertical patterns (see below). (For interpretation of the references to color in this figure legend, the reader is referred to the web version of this article.)

The approach applied in our study followed the basis of the Marine Modeling Framework (MMF), which is an integrated modeling tool developed at the Joint Research Centre to specifically assess the consequences of different scenarios on the ecosystem status of regional European Seas (Stips et al., 2015). The MMF includes the main elements of a Regional Earth System Model, i.e., the atmosphere, the hydrological basins and the oceans (Fig. 1).

The oceanic component of the MMF in its application to the Mediterranean Sea (Fig. 1) is composed of two coupled models, a 3D hydrodynamic model based on the General Estuarine Transport Model (GETM, Burchard and Bolding, 2002) and a biogeochemical model based on the Ecological Regional Ocean Model (ERGOM, Neumann, 2000), which were successively adapted to represent the Mediterranean ecosystems (MedERGOM, Macías et al., 2014a). The GETM implementation for the Mediterranean Sea (Fig. 1) has a horizontal resolution of 5′ × 5′ (∼9 × 9 km) and includes 25 vertical sigma layers. Model bathymetry was built using the ETOPO1 (http://www.ngdc.noaa.gov/mgg/global/) database, while the initial thermohaline conditions were created using the Mediterranean Data Archeology and Rescue-MEDAR/MEDATLAS database (http://www.ifremer.fr/medar/). The same MEDAR/MEDATLAS data were used to create the boundary conditions for the model at the Strait of Gibraltar, where monthly climatological vertically explicit values of salinity and temperature are imposed with no prescription for the horizontal currents at the open boundary.

GETM is forced at the surface every 6 h by the following atmospheric variables: wind velocity at 10 m, air temperature at 2 m, dewpoint temperature at 2 m, cloud cover and atmospheric pressure at sea level. Atmospheric data come from the European Centre for Medium-Range Weather Forecasts (ECMWF-ERA interim database). Bulk formulae were used to calculate the corresponding heat, mass and momentum fluxes between the atmosphere and the ocean (Macías et al., 2013).

The present configuration of the ocean model includes 53 rivers that discharge along the Mediterranean coast (red stars in Fig. 1) belonging to 8 river districts (black lines in Fig. 1). Values for river discharges were obtained from the Global River Data Centre (Germany) database, while inorganic nutrient loads (nitrate and phosphate) of freshwater runoff were obtained from Ludwig et al. (2009).

GETM was coupled online to the MedERGOM biogeochemical model (Macías et al., 2014a, Macías et al., 2014b) using the Framework for Aquatic Biogeochemical Models (FABM, Bruggeman and Bolding, 2014). MedERGOM is a modified version of the ERGOM model (Neumann, 2000) that is specifically adapted to represent the conditions of the pelagic ecosystem of the Mediterranean Sea. Briefly, MedERGOM incorporates three phytoplankton functional types (‘diatom-like’, ’flagellates-like’ and ‘cyanobacteria-like’), two major inorganic nutrients (nitrogen and phosphate), one zooplankton compartment and detritus. To obtain a more comprehensive description of this model, the reader is referred to Macías et al. (2014a) and Macias et al. (2018b). Initial biogeochemical and boundary conditions are computed from the World Ocean Atlas database (www.nodc.noaa.gov/OC5/indprod.html).

### 2.2 The ‘Line of Frugality’ concept

As stated earlier, the main aim of this contribution was to incorporate non-fixed nutrient uptake rates in the MedERGOM model to assess its suitability to reproduce Mediterranean ecosystems dynamics. MedERGOM equations for nutrients limitation are as follows:

(1) $$\mathrm{nlim}=\frac{{{(NH}_{4}+{\mathrm{NO}}_{3})}^{2}}{\alpha^{2}+{{(NH}_{4}+{\mathrm{NO}}_{3})}^{2}};$$

(2) $$\mathrm{plim}=\frac{{{\mathrm{PO}}_{4}}^{3-}}{{{i\_rfr}^{2}*\alpha }^{2}+{{\mathrm{PO}}_{4}}^{3-}};$$

*where nlim* and *plim* stand for ‘nitrogen limitation’ and ‘phosphate limitation’, respectively; NH_4_, NO_3_ and PO_4_^3−^ represent the concentration of ammonia, nitrate and phosphate in seawater, respectively (all in mmol/m^3^); α is the half saturation constant for N and P specific for each phytoplankton functional type included in the model and *i_rfr* is the P:N ratio in the organic matter.

In MedERGOM (as in ERGOM), the values of *nlim* and *plim* were computed for each position/time-step and for each phytoplankton type (ranging 0–1), with the lowest value selected as the nutrient-limiting factor.

In the original MedERGOM implementation, *i_rfr* was set at a fixed value of 1:16 following Redfield stoichiometry. This assumption implies that the molar composition of the organic matter (OM) in the original formulation was consistently 16N to 1P and that the limiting nutrient for phytoplankton growth was defined by the same ratio; accordingly, if the N:P_water_ was greater than 16N:1P, then phosphate limited production, whereas if the N:P_water_was below 16N:1P, then nitrogen was limiting. This approach is a common tool to simulate colimitation by different nutrients in low-complexity biogeochemical models that circumvents the need to fully consider the elementary composition of the OM and the different metabolic routes involved. It has, however, the disadvantage of its rigidity, which hampers consideration of the physiological adaptations of individual phytoplankton cells and/or community compositional changes in response to alterations in seawater chemical conditions.

In the LoF version of MedERGOM developed in this study, the assumption of a variable *i_rfr* is introduced to account for the plasticity of phytoplankton cells to take up phosphate at different rates depending on its ambient concentration in seawater. The exact formulation (Eq. (3) below) was derived from the N:C and P:C relationships provided by Galbraith and Martiny (2015), but in the particular case of the Mediterranean Sea, a constant N:C ratio of 144‰ was used, which is the mean value reported by Galbraith and Martiny (2015) for the range of nitrate concentrations measured in the basin (*e.g.,* Siokou-Frangou et al., 2010). A constant N:C ratio could introduce some error in the computation, albeit very small based on the reported range of variability for this ratio (between 140‰ and 150‰, as shown in Fig. 1 in Galbraith and Martiny, 2015). Hence, by dividing P:C (=6.9 * PO_4_^3−^ + 6(‰); according to Galbraith and Martiny, 2015) by N:C (=144 ‰), the P:N (*i_rfr* in Eq. (2) above) is simply computed as follows:

(3) $${i\_rfr}_{\mathrm{LoF}}=\frac{6.9*{\mathrm{PO}}_{4}^{3-}+6}{144};$$

where PO_4_^3−^ is the ambient water phosphate concentration in mmol/m^3^.

**Fig. 2:**
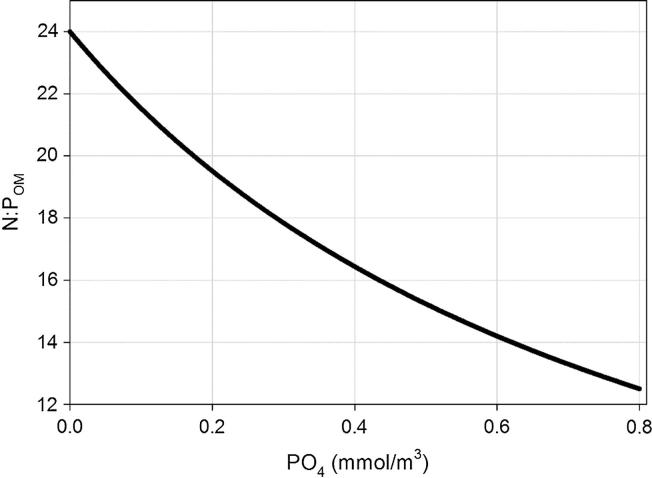
Dependence of the N:P_OM_ computed by the LoF formulation (Eq. (3)) versus the waters’ PO_4_ concentration.

With a variable ratio, changes in the OM stoichiometry (N:P_OM_) associated with particular nutrient features (i.e*.,* phosphate concentration) can be reproduced by the algorithm. Under phosphate scarcity, the phytoplankton-derived OM is expected to possess a higher-than-Redfield N:P molar ratio (up to a maximum of ∼24, Fig. 2), and conversely, limiting levels of N will result in a N:P_OM_ below Redfield. Consequently, this approach also has implications for determination of the limiting nutrient, as at each PO_4_^3−^ concentration in ambient water, the threshold for nitrate or phosphate limitation changes (according to Eqs. (1), (2)).

Implementation of the *i_rfr* defined in Eq. (3) thus allows the incorporation of phytoplankton flexibility for phosphate uptake under varying nutrient concentration conditions. Such deviations from the classical Redfield ratio may arise from physiological adaptations of phytoplankton cells in response to changes in environmental conditions or shifts in species compositions of the phytoplankton community due to variations in the preferred nutrient source (*e.g.,* Garcia et al., 2018).

In our study, two twin simulations covering the 2000–2007 period were performed, but only data from 2001 onwards were considered. The first simulation (RfR) assumed the classical Redfield ratio in the nutrient assimilation rate, whereas the second simulation (LoF) incorporated the LoF concept shown in Eq. (3). The remaining environmental conditions and physical forcing defined by the model parameters (initial values, boundaries, topography, etc.) did not differ among the two simulations.

## 3 Results

**Fig. 3:**
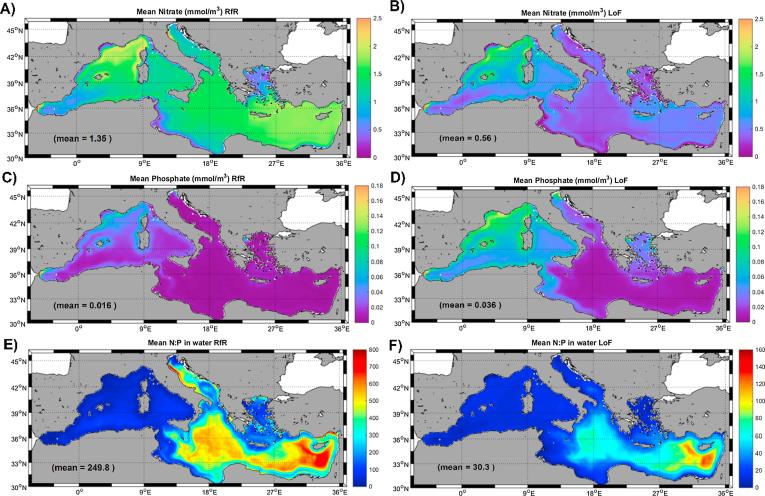
Mean climatological free nitrate and phosphate concentrations in seawater (0–10 m) for the RfR simulation (left column) and for the LoF simulation (right column). The climatological N:P ratio in water for each simulation are shown in the bottom panels. Inserted in each panel there is the mean, basin-wide integrated value of each represented variable. Please note the different color scales in panels E) and F). (For interpretation of the references to colour in this figure legend, the reader is referred to the web version of this article.)

Fig. 3 shows the mean climatological surface (0–10 m) seawater concentrations of the two major nutrients, nitrate and phosphate, in the Mediterranean Sea according to the two MedERGOM simulations. It is evident that phytoplankton nutrient uptake by a fixed or a variable N:P ratio affects nutrient content in the basin. A complete, comprehensive description of the simulated changes in the stoichiometry of nutrients in the Mediterranean waters can be found in Macías et al. (2018a). Therefore, our study focuses exclusively on the main differences found between the two model runs and briefly compares them with available nutrient data (Table 1) to stress the major consequences of the LoF implementation.

**Table 1:** Mean nutrients concentrations in regions A and B identified in Fig. 1 at different depth horizons from the RfR simulation, the LoF simulation and the reported range/average in the literature (Krom et al., 1991, Pasqueron de Fommervault et al., 2015, Powley et al., 2017 and references therein).

Region	Depth	NO_3_ (mmol/m^3^) RfR, LoF, (range in literature)	PO_4_ (mmol/m^3^) RfR, LoF, (range in literature)	N:P in water RfR, LoF, (average in literature)
A	Surface (0–100 m)	1.7, 1.2, (0–7.3)	0.04, 0.08 (0–0.35)	42.5, 15 (16.6)
	Deep (>1000 m)	8.0, 8.3, (1.6–9.5)	0.44, 0.48 (0.14–0.48)	18.2, 17.9 (20.8)

B	Surface (0–100 m)	1.8, 0.8, (0.01–1.5)	0.007, 0.018 (0–0.1)	503, 74 (90)
	Deep (>1000 m)	4.6, 4.7, (3–6)	0.16, 0.17 (0.13–0.23)	28.7, 28.3 (28.5)

Regarding nitrate concentrations (Fig. 3A and B), the LoF run clearly resulted in mean surface values across the entire basin that were much lower than those obtained with the RfR simulation (0.56 mmol/m^3^ vs 1.13 mmol/m^3^). The spatial distribution of nitrate with an Redfield stoichiometry exhibited an evident north-south gradient, with slight differences between the western and eastern basins (maximum mean surface concentrations of ∼2 mmol/m^3^ in both regions). In contrast, surface nitrate with a LoF assumption was scarcer in the eastern basin (maximum concentration ∼0.5 mmol/m^3^) than in the western basin (maximum ∼1.8 mmol/m^3^) where a certain north-south gradient was still present.

Regarding phosphate, an opposite pattern was found (Fig. 3C and D), as the mean surface concentration was higher in the LoF simulation (∼0.036 mmol/m^3^) than in the RfR run (∼0.014 mmol/m^3^). A similar north-west to south-east gradient could be observed in both distribution maps with some large differences in marginal basins such as the Adriatic, the Aegean and the Gulf of Gabes region, where phosphate levels markedly increased with the LoF assumption (Fig. 3D). Mean phosphate values obtained in both runs were within the reported range of observations (Table 1); therefore, a definitive conclusion concerning the suitability of each simulation to better reproduce in situ values could not be drawn.

As a consequence of the patterns described above, the N:P ratios in seawater derived from each simulation were clearly different. Mean N:P_water_ resulted in a value of ∼250 assuming a Redfield stoichiometry in phytoplankton nutrient uptake, whereas the LoF concept provided a mean value of ∼30. Nevertheless, in both cases, an increasing gradient towards the south-east was evident (see Fig. 3E and 3F), with the N:P_water_ reaching the highest values in the easternmost region of the basin (∼700 in RfR and ∼100 in LoF). Compared with the observational patterns of the N:P ratio, both the mean values and spatial distribution were closer to the LoF outputs (Table 1). In fact, the combination of elevated nitrate concentrations with very low phosphate levels under an RfR assumption resulted in unrealistically large values for the N:P ratio in surface waters of the Mediterranean Sea (Table 1).

**Fig. 4:**
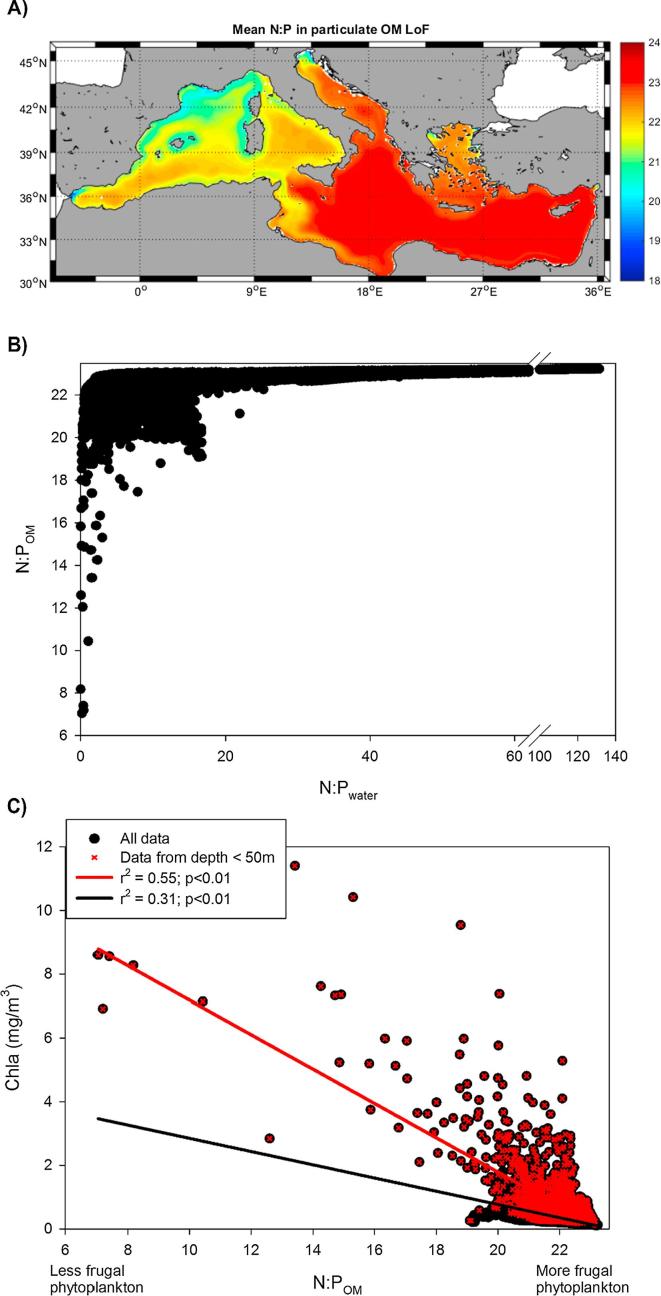
(A) Mean climatological molar N:P ratio in organic matter (OM) for the LoF simulation. (B) Scatter plot of N:P in the organic matter versus N:P in water for the LoF simulation. (C) Scatter plot of chlorophyll concentration versus N:P ratio in the organic matter for the LoF simulation.

The effects of using the LoF formulation not only altered the free nutrient levels in water (Fig. 3) but also the composition of the particulate organic matter (Fig. 4A). A north-west to south-east increasing trend was clearly visible with a mean N:P_OM_ ratio of approximately 22.4 for the entire basin, which is clearly higher than Redfield. When the N:P_OM_ spatial distribution was compared to that of N:P_water_ (Fig. 3F), a positive correlation between both ratios was found (Fig. 4B). There was, however, a deviation of this general pattern at N:P_water_ ∼16 (the Redfield ratio), wherein a small cloud of data demonstrated the opposite trend. The relationship between the Chla concentration and the N:P_OM_ value showed a negative correlation (Fig. 4C), which became particularly evident and significant when only coastal regions (<50 m depth) were considered (red line and dots in Fig. 4C). This finding suggested that the adoption of a variable N:P ratio was more suitable to describe coastal populations patterns than open-sea phytoplankton communities.

**Fig. 5:**
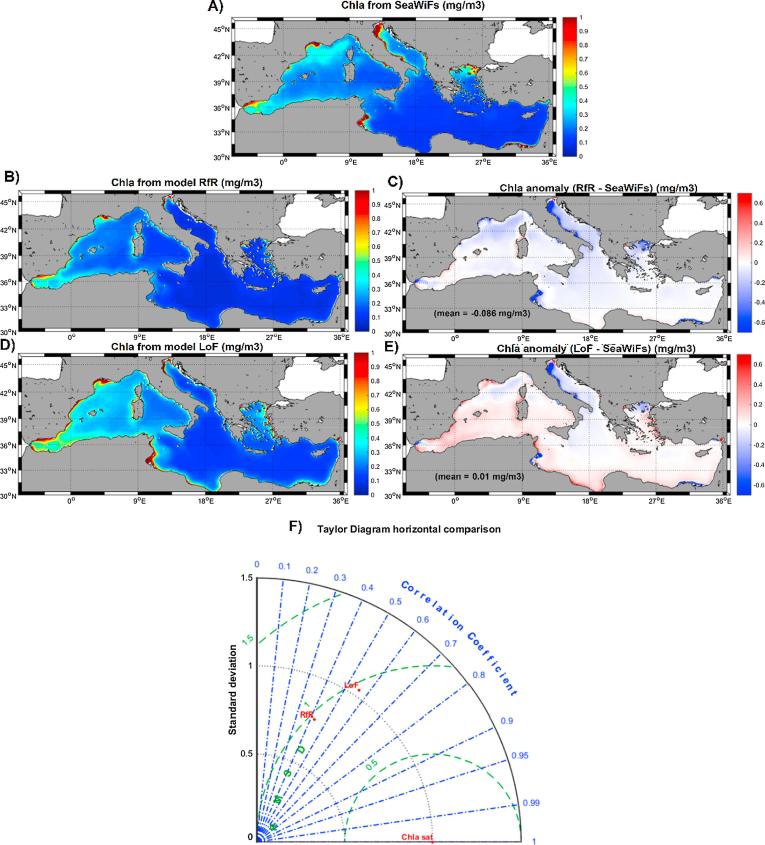
Satellite and model climatological surface chlorophyll for the period 2001–2007. (A) SeaWiFs mean surface chlorophyll concentration (mg/m^3^). (B) RfR simulation mean surface chlorophyll concentration (mg/m^3^). (C) Mean RfR – SeaWiFS surface chlorophyll anomaly (mg/m^3^). (D) LoF simulation mean surface chlorophyll concentration (mg/m^3^). (E) Mean LoF – SeaWiFS surface chlorophyll anomaly (mg/m^3^). (F) Taylor Diagram of model-satellite surface chlorophyll comparison.

The effect of adopting a differential phosphate uptake rate on the distribution of the surface phytoplankton biomass was subsequently assessed at the basin scale. The analysis was performed by comparing the model results with independent estimates of phytoplankton biomass obtained through satellite-derived chlorophyll (Chla), despite some issues associated with remote sensing Chla estimates for the Mediterranean Sea, as discussed further below. Fig. 5 shows the surface climatological (2001–2007) Chla distribution maps obtained by remote sensing (panel A, computed from http://oceancolor.gsfc.nasa.gov) by the MedERGOM RfR run (panel B) and the MedERGOM LoF run (panel D), as well as the anomaly maps (model – satellite) in panels C and E.

**Fig. 6:**
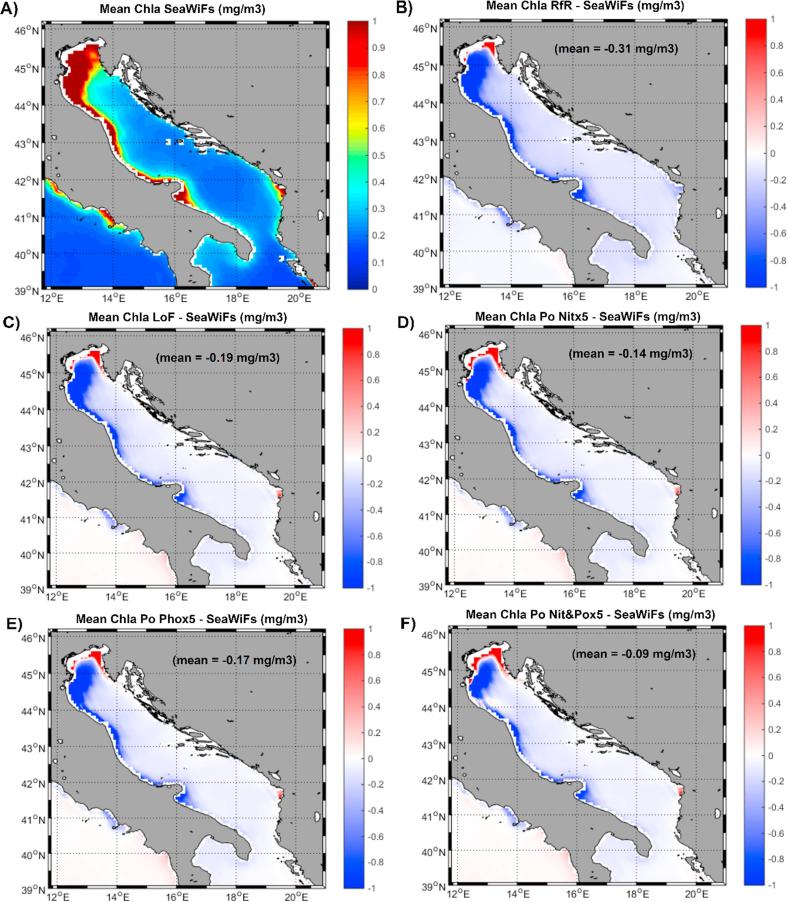
(A) Climatological (2001–2007) surface chlorophyll from SeaWiFs (mg/m^3^). (B) Anomalies (model – satellite) of surface chlorophyll for the RfR run (mg/m^3^). (C) Anomalies (model – satellite) of surface chlorophyll for the LoF run (mg/m^3^). (D) Anomalies (model – satellite) of surface chlorophyll for the LoF run increasing nitrate concentration in the Po River five times (mg/m^3^). (E) Anomalies (model – satellite) of surface chlorophyll for the LoF run increasing phosphate concentration in the Po River five times (mg/m^3^). (F) Anomalies (model – satellite) of surface chlorophyll for the LoF run increasing nitrate and phosphate concentrations in the Po River five times (mg/m^3^).

**Fig. 7:**
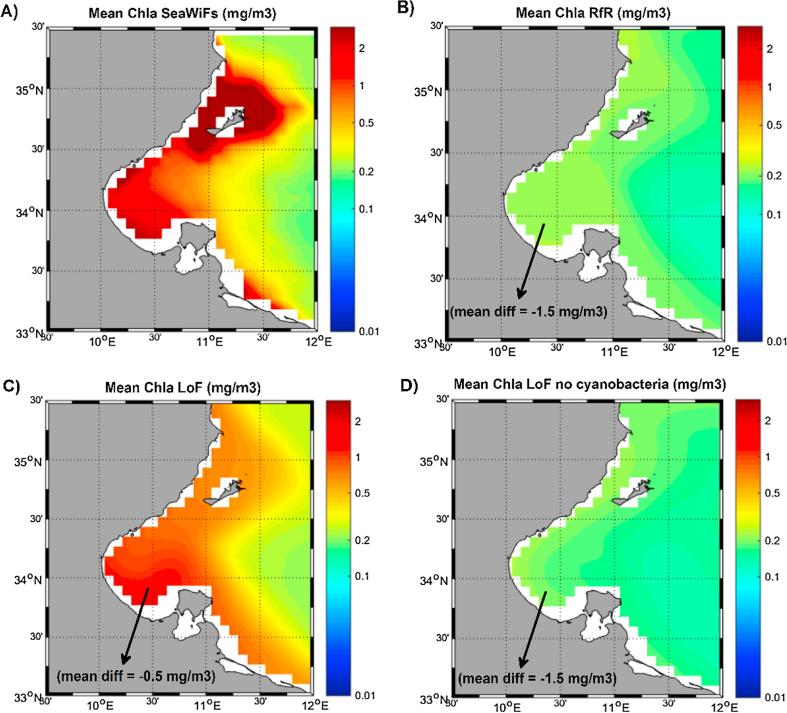
(A) Climatological (2001–2004) surface chlorophyll from SeaWiFs (mg/m^3^). (B) Climatological (2001–2007) surface chlorophyll from RfR run (mg/m^3^). (C) Climatological (2001–2007) surface chlorophyll from LoF run (mg/m^3^). (D) Climatological (2001–2007) surface chlorophyll from LoF run eliminating the cyanobacteria group (mg/m^3^).

Even though the general distribution pattern coincided in the three distribution maps (Fig. 5A, B and D), with the usual west-east oligotrophy gradient identified regardless of the approach, the RfR simulation failed to fully reproduce productivity ‘hotspots’ known to occur in some coastal regions, such as the Alboran Sea, the Gulf of Lions, the Adriatic and Aegean Seas and the Gulf of Gabes. In addition, the mean basin-wide Chla obtained from satellites and equivalent to 0.236 mg/m^3^ matched quite well the average modeled Chla level provided by the LoF simulation (0.246 mg/m^3^), whereas the RfR run yielded an average Chla concentration of 0.151 mg/m^3^. Because of the discrepancies observed in coastal areas between the two model approaches, the mean anomaly of modeled surface Chla in relation to satellite estimates markedly decreased in the LoF simulation (Fig. 5E, mean anomaly = 0.01 mg/m^3^) with respect to that obtained with the RfR run (Fig. 5C, mean anomaly = −0.086 mg/m^3^). The Taylor diagram (Fig. 5F) unequivocally confirmed that the surface Chla distribution at the basin scale obtained with the LoF run better mimicked the satellite estimates, as both the correlation coefficient and standard deviation were more concordant in this case than applying the RfR simulation. Fig. 6, Fig. 7 focus on two particular coastal areas where RfR simulation failed to simulate the relatively high surface Chla levels detected by remote sensing in the Adriatic Sea and the Gulf of Gabes (GoG). In both regions, inclusion of the LoF concept reduced the model bias from −0.31 mg/m^3^ to −0.19 mg/m^3^ in the Adriatic Sea (Fig. 6B and C) and from −1.5 mg/m^3^ to −0.5 mg/m^3^ in the GoG (Fig. 7B and C).

The time-series of basin-integrated surface Chla values are shown in Fig. S1A for the SeaWiF estimates and the two model runs. The Chla temporal variability displayed the same seasonality regardless of the approach considered, with the typical late-winter, early-spring bloom in the basin, very low values during the stratification period and a progressive increase during the fall. The intensity of the phytoplankton bloom seemed to be overestimated by the LoF run, although during the rest of the year, the model and satellite Chla data matched quite well, with a very high correlation coefficient (∼0.87) and similar standard deviations (Fig. S1B). In contrast, Chla simulated with the RfR run was lower than the satellite estimates throughout the year, although the correlation coefficient between the two series was very high (∼0.95) and the modeled data presented a lower standard deviation than the remote sensing estimates (Fig. S1B).

Based on previous modeling studies (Macías et al., 2014b), it has become clear that the vertical distribution of the planktonic community is a very important factor determining the total basin productivity in the Mediterranean Sea. This parameter is especially relevant during the stratification period when surface Chla levels are very low throughout the basin but subsurface Chla accumulations are ubiquitous and relatively large. Thus, a subsequent analysis was conducted to examine whether the inclusion of a variable N:P ratio in the biogeochemical model had any effect on the vertical distribution of the phytoplankton biomass at a basin scale. Fig S2 depicts the vertical profiles of chlorophyll obtained with the two model runs in the two open-water regions plotted in Fig. 1 and that have been previously examined by Macías et al., 2014b, Macías et al., 2018a.

In region A of the western basin (Fig. S2, left column), the vertical distribution of Chla showed similarities between both model runs (Fig. S2A and S2C), with a late-winter bloom occupying the upper 50 m of the water column and subsequently developing to a deep chlorophyll maximum (DCM) during the stratification period. The DCM in July was more robustly simulated in the RfR than in the LoF simulation (75 m vs 61 m, respectively), which also revealed higher concentrations in the latter case. However, in general, LoF simulates larger surface Chla values, which decrease at depths below 100 m during the stratification period (Fig. S2E). For region B in the eastern Mediterranean (Fig. S2, right column), the effect of applying a LoF concept on the Chla vertical distribution resulted in a slight delay of the winter bloom, which occurred in March instead of February as in the case of a Redfield assumption. Moreover, the DCM during the stratification period was larger in the LoF simulation, with a position in the water column that was almost identical in both approaches (∼93 m depth). Accordingly, differences between both model runs at site B were positive up to a depth of 150 m (Fig. S2F).

**Fig. 8:**
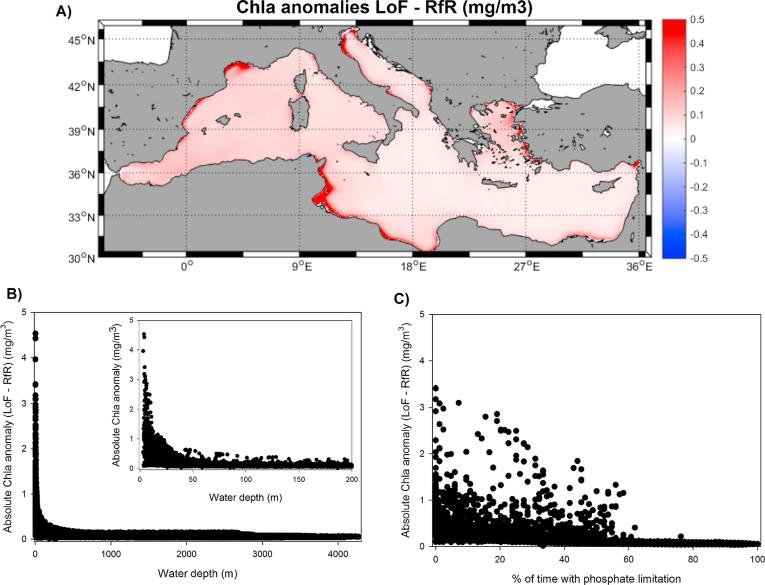
(A) Mean difference in surface chlorophyll between RfR and LoF simulations (mg/m^3^). (B) Scatter plot the absolute difference in surface chlorophyll between RfR and LoF versus water depth. (C) Scatter plot the absolute difference in surface chlorophyll between RfR and LoF versus the percentage of phosphate limitation.

When the analysis of the Chla anomaly between both model runs was extended to the basin scale, the LoF assumption clearly resulted in higher mean values throughout almost the entire basin (Fig. 8A), and the magnitude of the anomaly was negatively related to the water depth, as maximum differences were found in shallow regions (Fig. 8B).

**Fig. 9:**
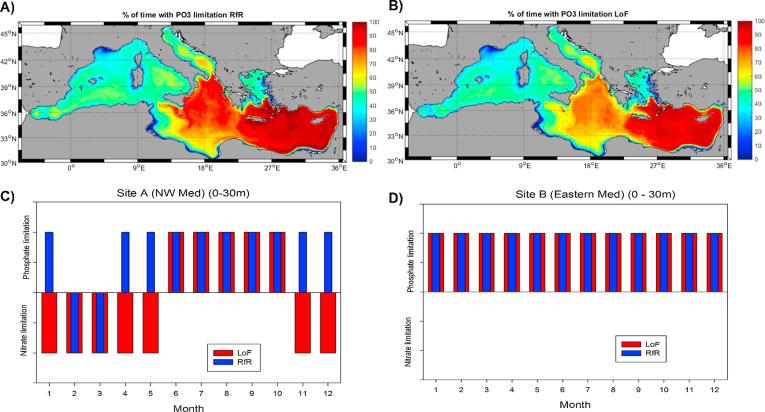
(A) Map of the percentage of time with phosphate limitation for the RfR simulation. (B) Map of the percentage of time with phosphate limitation for the LoF simulation. (C) Climatological seasonal cycle of nutrient limitation at site A (see Fig. 1) for RfR (blue bars) and LoF (red bars). D) as panel C) but for site B (see Fig. 1). (For interpretation of the references to color in this figure legend, the reader is referred to the web version of this article.)

As the LoF version of the MedERGOM satisfactorily reproduced the spatial and temporal dynamics of surface and subsurface Chla in the basin, model simulations were also used to assess the extent of phosphate limitation on primary production in different regions. Using the ambient nutrient concentrations simulated by each model run (Fig. 3) and the model equations defining nutrient limitation conditions (Eqs. (1), (2)), the period of time in which phosphate constrained production was computed at each grid position (Fig. 9A and B). Despite the observed differences in the distribution of nutrients in surface waters (Fig. 3), production seemed to be limited by phosphate during similar periods for the two simulations (Fig. 9): ∼62.4% of the time for RfR and ∼57.8% of the time for LoF. Furthermore, both model runs marked a clear west-east gradient, with the eastern basin almost always being phosphate limited (90% of the time). Exceptions to this general pattern were found in the Adriatic and Aegean Seas, where phosphate limitation in terms of time period decreased in the LoF simulation.

In the two regions considered above (A and B, Fig. 1), the analysis was extended to both nutrients for each climatological month. Fig. 9C shows that with a LoF assumption, nitrate limits phytoplankton proliferation during late fall, winter and spring in the NW Med (Fig. 9C, red bars), whereas with a Redfield stoichiometry, nitrate limitation only proceeds (on climatological scale) during February and March (Fig. 9C blue bars). During the stratification period (May to October), both simulations indicated that phosphate was the limiting nutrient for phytoplankton growth. Similarly, both runs showed a permanent phosphate limitation in surface waters of the Eastern Mediterranean (Fig. 9D).

Moreover, a negative relationship between the Chla anomaly between both model simulations and the time length under phosphate limitation was observed (Fig. 8C). When phosphate was the major limiting nutrient (>60% of the time), the differences in Chla estimated between LoF and RfR runs were negligible, but where phosphate limitation was reduced (<60% of the time), the Chla differences became noticeable. Thus, this plot indicates that the effect of using a variable N:P ratio in phytoplankton uptake is only noticeable when the phosphate limitation is not overwhelmingly strong.

## 4 Discussion

The main consequences of the adoption of the LoF concept for the stoichiometry of nutrients in the Mediterranean Sea are decreases in the levels of free nitrogen in surface waters and a slight increase in surface phosphate concentrations, which are patterns that were not observed with the usual assumption of a Redfieldian uptake rate (Fig. 3). Comparison with available data (Table 1) and with the extensive report by Macías et al. (2018a) indicated that surface nitrate levels obtained in the RfR simulation were too high (∼1.8 mmol/m^3^ on average), especially in the Eastern Mediterranean, in relation to the in situ reported values ranging from 0.3 to 0.7 mmol /m^3^ (*e.g.*, Krom et al., 1991, Powley et al., 2017). Based on historical nitrate measurements covering the period from 1961 to 2010, Pasqueron de Fommervault et al. (2015) showed that surface levels in the Levantine basin never exceeded 0.5 mmol N/m^3^, which reinforced that the RfR version of the MedERGOM clearly overestimated the surface nitrate concentration in this region. It must be stressed that nutrient measurements in Mediterranean Sea oligotrophic waters are problematic. Many of the samples used for the data reported in Table 1 were frozen unfiltered until the time of measurement, and it has been shown (Krom et al., 2005) that this procedure introduces large random errors when N and P concentrations are below ∼400 nM and below ∼ 20 nM, respectively (which represents most of the Mediterranean). Hence, to better understand the nutrient dynamics in this particular basin and to calibrate/validate biogeochemical models like the one presented herein, a consistent methodology for sampling, storing and analyzing nutrient samples should be established and observed in all future research efforts in this particular marine basin.

As a result of changes in individual nutrient concentrations, the modeled N:P_water_ ratio in surface waters varied appreciably between both simulations (Fig. 3E and 3F). Accordingly, the mean RfR value (∼250) was disproportionally high in relation to field measurements (*e.g.*, Krom et al., 1991, Pasqueron de Fommervault et al., 2015, Powley et al., 2017), whereas the mean N:P_water_ value obtained with the LoF assumption (∼30) mimicked previously reported ratios.

In addition, the modeled N:P_OM_ ratio increased from west to the east following (inversely) the phosphate limitation gradient (Fig. 4A). This increase in the internal nutrient ratio with decreasing phosphate levels is a direct consequence of the implementation of the LoF approach (Eq. (3)) and agrees with the notion that phytoplankton can be economical with their phosphorous requirements when the supply is constrained (*e.g.,* DeVries and Deutsch, 2014). It also agrees with the findings of Tanaka et al., 2011, Krom et al., 2014 based on field data for the nitrate and phosphate contents in the organic matter of the Mediterranean Sea and with the theoretical map reported by Galbraith and Martiny (2015). The maximum value of N:P_OM_ obtained herein (∼24) was much lower than those reported by Fransner et al. (2018) in the northern Baltic Sea (maximum value ∼150) using a coupled 3D physical-biogeochemical model that considered a non-Redfield stoichiometry. It is important to stress that the LoF implementation yielded a maximum N:P_OM_ of 24 (corresponding to a phosphate concentration of 0, Fig. 2) and that the value obtained in Baltic waters could be overestimated, as recognized by the authors (maximum N:P_OM_ measured in the Gulf of Bothnia of ∼21 according to Pertola et al., 2002). Nevertheless, our study findings are consistent with the general conclusions drawn by Fransner et al. (2018) in the Baltic, as phytoplankton stoichiometric flexibility in particular, with a Lof concept, is key to simulate nutrient dynamics and seasonal Chla in the Mediterranean Sea. If the constant Redfield ratio was instead used to reproduce spatiotemporal patterns of both biogeochemical variables or N:P_OM_ in the basin, as assumed in most biogeochemical models currently in use, the MedERGOM outputs did not fully reflect the observed trends, particularly in coastal areas.

The negative correlation found between N:P_OM_ and Chla in the LoF simulation (Fig. 4C) indicated that more ‘frugal’ phytoplankton (as defined by Galbraith and Martiny, 2015) were associated with lower Chla levels, while less ‘frugal’ species were able to thrive much faster and reach higher Chla concentrations. This result is in line with the ‘*growth rate hypothesis’* (Sterner and Elgar, 2002) that states that the N:P ratio is expected to decline with an increasing growth rate (Hillebrand et al., 2013), even if the Chla concentration is not fully equivalent to the growth rate. It must also be indicated that in MedERGOM, higher Chla values typically correspond to the ‘diatom-like’ functional group (Macías et al., 2014b), which is known to display faster growth rates but lower plasticity with respect to phosphate requirements (Ho et al., 2003, Quigg et al., 2003). This lower plasticity to phosphate would explain the occurrence of diatom blooms in mesotrophic areas of the Mediterranean Sea, where ‘new-nutrients’ are usually supplied to the photic layer, such as the western Alboran Sea or the Gulf of Lions (*e.g.,* Macías et al., 2006, Severin et al., 2014).

The injection of new nutrients in mesotrophic regions results in deviations in the negative relationship between N:P_water_ and N:P_OM_ (Fig. 4B) (see Macías et al., 2018a and Fig. S3 for an identification of the ‘anomalous’ points: the Alboran Sea and the NW Mediterranean region). In the Alboran Sea, allochthonous nutrients are supplied to the photic zone by the incoming Atlantic waters (Minas et al., 1991, Béthoux et al., 1998, Huertas et al., 2012) and the interfacial mixing induced by tidal dynamics within the Strait of Gibraltar (Macías et al., 2007). In the NW Mediterranean, the meandering northern current (*e.g.,* Oguz et al., 2015) and deep water convection events (Macías et al., 2018b) deliver nutrients to the upper layer, thereby favoring new production and altering the general oligotrophic conditions of the basin. In both regions, the relationship N:P_water_ vs N:P_OM_ is positive (Fig. S3), and the mean N:P_water_ equals ∼16 (i.e., the Redfield ratio value).

Other factors clearly influence the N:P_OM_ in plankton, such as light and other nutrients (Goldman, 1986), the internal nutrient quota (Klausmeier et al., 2004), or even the physiological status and past limitation history of phytoplankton populations (Hillebrand et al., 2013), which are not included in our low-complexity biogeochemical model. However, this LoF approach, even with its simple formulation, is able to represent some of the observed relationships between ambient water/internal nutrient ratios and between fast/slow growing phytoplankters (Hillebrand et al., 2013), which is an encouraging indicator of the potential application of this approach more broadly in other marine regions.

Nevertheless, changes in free surface nutrient levels and organic matter stoichiometry did not have a dramatic impact on the phytoplankton biomass in surface waters. As shown in Fig. 5, adopting the LoF approach generally increased the amount of surface chlorophyll, especially in the Alboran Sea, the northern Adriatic, the Aegean Sea and the GoG, thus bringing the model simulations closer to the satellites estimates (Fig. 5F). The underestimation of surface chlorophyll in the northwestern coast of the Adriatic Sea and in the northern Aegean Sea with respect to satellite values is a common issue in biogeochemical models for the Mediterranean Sea (*e.g.,* Lazzari et al., 2012, Lazzari et al., 2014, Macías et al., 2014a, Macías et al., 2014b, Guyennon et al., 2015, Richon et al., 2018, Tsiaras et al., 2017). Our study findings showed that inclusion of the LoF approach in the MMF clearly reduced discrepancies with model-satellite estimations in those regions (Fig. 5E), lowering the mean bias from −0.31 mg/m^3^ to −0.19 mg/m^3^ in the Adriatic and from −0.12 mg/m^3^ to -0.06 mg/m^3^ in the Aegean Sea. Differences between remote sensing data and LoF outputs for the Aegean Sea can be considered very low given the difficulty related to properly simulating the complex dynamics of this basin (Petihakis et al., 2014) with a relatively coarse resolution such as that used in the present MMF implementation. However, in the case of the Adriatic Sea, the underestimation of the phytoplankton biomass on its western coast in particular could, potentially, have another origin.

In this subbasin, the major source of allochthonous nutrients to the water column are the River Po discharges (*e.g.,* de Wit, 2001, Justic et al., 1995, Relevante and Gilmartin, 1973). Therefore, a plausible reason for the observed mismatch between the satellite estimates and model simulations might lie on the imposed nutrient loads from the river. To assess this possibility, three new simulations with the LoF version of MedERGOM were performed for the 2000–2007 period by changing the nutrient concentrations in River Po waters: 5× nitrate; 5× phosphate and 5× nitrate and phosphate (Fig. 6D–F). As described above, the inclusion of the LoF formulation reduced the bias with respect to the satellites by almost half its value with the RfR approach (see Fig. 6B and C); increasing the nitrate concentration five times in the river discharge only reduced the overall bias to −0.14 mg/m^3^ (Fig. 6D) and increasing the phosphate concentration results with a mean bias of −0.17 mg/m^3^ (Fig. 6E). Nevertheless, when both inorganic nutrients were contemporarily increased in the river inputs (Fig. 6F), the bias compared with satellite estimates clearly diminished to −0.09 mg/m^3^.

However, manipulating the Po nutrient loads mainly affected the phytoplankton biomass in the northern shelf of the Adriatic Sea, which is in the immediate vicinity of the river mouth. The elongated tongue of high Chla levels spotted by remote sensing along the eastern Italian coast was not resolved by any of the model runs. Thus, the lack of simulated chlorophyll in this coastal fringe is unlikely to be related to Po river fertilization and may be attributable to other processes, such as circulation mechanisms. The relatively coarse spatial resolution of the model would not allow a correct representation of the specific hydrodynamic processes occurring in coastal regions (Wolanski and Hamner, 1988). Moreover, the atmospheric forces used herein might not be able to correctly represent the wind patterns around complex topographies, such as those present in this particular region of the basin (Macías et al., 2014a). In addition, an alternative and complementary reason for the observed discrepancies could be related to the intense anthropogenic pressure suffered by this coastal region through activities that include tourism, gas/oil extraction, intensive fishing and fish farming (ADRIPLAN), which are not included in the MMF but undoubtedly alter the environmental status of the marine ecosystem in the area.

Another region in which biogeochemical models subestimated the surface chlorophyll concentration with respect to satellite estimates is the Gulf of Gabes (GoG). Intense and quasi-constant satellite-estimated phytoplankton blooms were typically detected in the gulf (see Fig. 5A). In fact, remote-sensing images for this region revealed two different ‘hotspots’: one around the Kerkennah Islands and another in the inner zone of the gulf (Fig. 7A). Satellite estimates in the former region (around the islands) could be affected by artifacts linked to very highly turbid waters near the coast and the presence of seagrass meadows surrounding the archipelago (*e.g.*, Jaquet et al., 1999). However, the bloom inside the GoG has been measured in situ (*e.g.,* Sabeur et al., 2016) and associated with the proliferation of cyanobacteria (*e.g.,* Loukil-Baklouti et al., 2018). The LoF simulation revealed the substantial biological production in the GoG area but not around the islands, as illustrated in the chlorophyll map in Fig. 7C. To the best of our knowledge, this is the first study to present a biogeochemical model designed for the whole Mediterranean Sea that is able to reproduce significant primary production levels in this coastal region and a first-order indication of the need to incorporate flexible internal nutrient quotas in biogeochemical models to achieve a correct representation of this marginal sea (Richon et al., 2018). The persistent significant underestimation of Chla levels simulated with the LoF approach within the GoG (∼0.5 mg/m^3^ on average, Fig. 7C) could be related to the lack of modeled nutrient sources in the region, such as the pollution derived from the intense industrial production of phosphoric acid (*e.g.,* El Kateb et al., 2018).

To further assess the importance of N-fixing organisms for the chlorophyll pattern in the GoG, a final simulation with the LoF version of MedERGOM was performed, but in this case, the cyanobacteria group was eliminated from the algorithm by simply imposing a negligible maximum growth rate for this phytoplankton group. As a result, the chlorophyll maximum in the GoG disappeared (see Fig. 7D), with a bias with respect to the satellite data rising to the same level as with the application of the RfR model (−1.5 mg/m^3^). It is worth mentioning that the chlorophyll simulated in this region with the LoF run (Fig. 7C) included the three different phytoplankton functional types (cyanobacteria, flagellates and diatoms). Henceforth, the attenuation of the surface chlorophyll concentration in the inner part of the gulf following the removal of cyanobacteria from the model (Fig. 7D) could be partially attributed to the absence of this phytoplankton group, but the remaining phytoplankton groups were clearly also affected.

Chla seasonality in the whole basin was well captured by the two model runs (Fig. S1A), with the typical winter-spring bloom evidenced along with the oligotrophy that occurs during the stratification period (*e.g.,* Siokou-Frangou et al., 2010). Although the value of the correlation coefficient was higher for the RfR simulation (r^2^ ∼ 0.95) than for the LoF simulation (r^2^ ∼ 0.87), the former showed a larger mean bias (∼−0.07 mg/m^3^) than the latter (0.01 mg/m^3^). In terms of relative error (|data-model|/data), differences were 0.33 and 0.058 for RfR and LoF, respectively. The RfR relative error was within the range reported by Tsiaras et al. (2017) (0.21–0.42 using different data assimilation methods), whereas the smaller LoF relative error indicated better fitting of the model with satellite estimates using this approach.

Furthermore, the vertical distribution of Chla concentration was also affected by implementation of the LoF approach. DCMs are known to be ubiquitous features in the Mediterranean Sea (Siokou-Frangou et al., 2010, Oguz et al., 2013), especially during the stratification period (Macías et al., 2014b). Both model runs showed DCMs in the two open-sea analyzed regions (Fig. S2) but with varying intensity. With the adoption of the LoF, not only did the surface winter/spring blooms appear more intense, but there was also an increase in Chla values within the DCMs during the stratified season.

If we compare our simulations for the Eastern Mediterranean (site B, Fig. S2 – right column) with the data reported by Krom et al. (1991), the LoF winter bloom (∼0.22 mg/m^3^) matched their observed value equivalent to ∼0.25 mg/m^3^. In addition, the DCM intensity in summer was ∼0.17 mg/m^3^ in the LoF run, in agreement with that measured in situ at a depth of 90–100 m (where the model also situated the DCM) and corresponding to ∼0.2 mg/m^3^. For both features (surface winter bloom and summer DCM), the RfR run provided lower Chla levels (Fig. S2F). In the case of the NW Mediterranean, the vertical Chla distribution obtained with both model runs (site A, Fig. S2 – left column) coincided roughly with previous observations in the region (Dolan and Marrasé, 1995), with a summer DCM at a depth of ∼60–70 m. Nevertheless, Chla values at the DCM level provided by the LoF approach were almost two-fold higher than those resulting from the RfR simulation (∼0.4 mg/m^3^ compared with 0.22 mg/m^3^) and closer to the reported values (Dolan and Marrasé, 1995).

Our approach also evidence the major limiting role of phosphate in the Mediterranean Sea (*e.g.,* Krom et al., 1991, Kress and Herut, 2001, Lazzari et al., 2016) (Fig. 9A and 9B), with mean percentage values for the limitation time of ∼62.4% and ∼57.8% in the RfR and LoF simulations, respectively. As described elsewhere (*e.g.*, Owen et al., 1989; Krom et al., 2010, Tanaka et al., 2011, Richon et al., 2018), phosphate-limited phytoplankton productivity exhibits a west to the east gradient. In the western basin, nitrate and phosphate were colimiting, with phosphate limitation represented between 25 and 45% of the time, with some exceptions at coastal sites with a strong riverine influence (such as the inner part of the Gulf of Lions), where nitrate became the limiting nutrient (Fig. 9). As we moved eastwards, phosphate limitation arose in both model runs and reached 100% of the time in the Eastern (*e.g.*, Krom et al., 1991, Krom et al., 2010, Powley et al., 2017). We must stress here that we are examining monthly mean snapshots of the model simulation and that shorter-term limitations could look different, as indicated by Thingstad et al. (2005), who found N and P colimitation in the Eastern Mediterranean during certain periods of the summer. Additional noteworthy exceptions are coastal areas, such as the Gulf of Gabes, the vicinity of the Nile outlet and the Aegean Sea, where significant nitrate limitation is simulated by the model using both approaches.

In terms of the climatological seasonality of nutrient limitations, the west to the east gradient could also be observed (Fig. 9C and D). For the NW Mediterranean, both model runs reported a shift in the limiting nutrient (Fig. 9C). It has been reported that nitrate becomes limiting during late-fall, winter and spring in this subbasin (*e.g.,* Tanaka et al., 2011, Powley et al., 2017, Richon et al., 2018), in accordance with the pattern shown by the LoF simulation (red bars in Fig. 9C). The RfR run, in contrast, indicated only that nitrate limited biological production in the NW Mediterranean during the months of February and March (blue bars, Fig. 9C). For the eastern basin (Fig. 9D), both model runs indicated that phosphate consistently limited primary production, in good agreement with former observations (*e.g.*, Krom et al., 1991).

The back to back comparison of the simulated Chla in the RfR and LoF runs provides interesting insights concerning the effects of the use of variable internal nutrient ratios. The first clear effect is that, under identical conditions, the LoF simulation resulted in higher levels of surface chlorophyll almost everywhere in the basin (Fig. 8A, mean bias ∼0.09 mg/m^3^), but especially along the coast, where climatological Chla differences could be as high as 3–4 mg/m^3^ (Fig. 8B). It is also remarkable that the largest bias was attained in those regions where phosphate limitation was lower and nitrate co-limitation was present (Fig. 8C). This finding indicates that the addition of flexibility to the internal nutrient quota presents an advantage under conditions of nutrient co-limitation but has minimal effects when phosphate is clearly the limiting resource (i.e., when production is restricted by phosphate >50% of the time).

## 5 Conclusions

In general, the adoption of an adjustable internal N:P ratio in the MedERGOM biogeochemical model provided a better description of the stoichiometric nutrient conditions in Mediterranean Sea waters. By using the LoF assumption, the concentration of both nutrients in seawater, especially their relative ratio, were in better agreement with previous field reports and in situ measurements. Thus, mean absolute biases for N:P in surface waters were 25.9 and 413 for the RfR (points A and B) and were reduced to 1.6 and 16 for the LoF (points A and B).

This strategy also improved the fitting between simulated surface chlorophyll and remote-sensing chlorophyll estimates. The mean spatial bias was reduced from −0.086 mg/m^3^ in RfR to −0.01 mg/m^3^ in the LoF, while mean temporal differences declined from −0.075 mg/m^3^ in RfR to −0.031 mg/m^3^ in LoF. This improvement was especially evident in certain coastal sites, such as the GoG (bias reduced from −1.5 to −0.5 mg/m^3^), the northern Adriatic (from −0.31 to −0.15 mg/m^3^) and the Aegean Seas (from −0.12 to −0.06 mg/m^3^), where whole-basin biogeochemical models typically experience difficulties in reproducing satellite-derived chlorophyll levels. Furthermore, vertically, an improvement in the fitting between simulated and measured subsurface chlorophyll accumulation (both in terms of the simulated concentration and position of the maximum) was observed when the LoF approach was adopted.

In summary, although far from providing a completely satisfying description of the pelagic ecosystem, the adoption of variable, non-Redfield N:P dynamics in a simple biogeochemical model significantly improved the fitting between the available data and the simulations results. As this comes at a null cost in terms of computational model time (difference for one-month simulation of ∼0.027 sec), this is an appealing approach that should be considered for other similar models being applied to this particular marginal sea and to other regions with a non-Redfieldian stoichiometry.
