# Supplementary material for: Non-Redfieldian dynamics driven by phytoplankton phosphate frugality explain nutrient and chlorophyll patterns in model simulations for the Mediterranean Sea
Source: Prog Oceanogr. 2019 Apr;173:37–50. doi: 10.1016/j.pocean.2019.02.005 (PMC7099761; doi:10.1016/j.pocean.2019.02.005)
Supplement: Supplementary data 2 [file mmc2.docx]

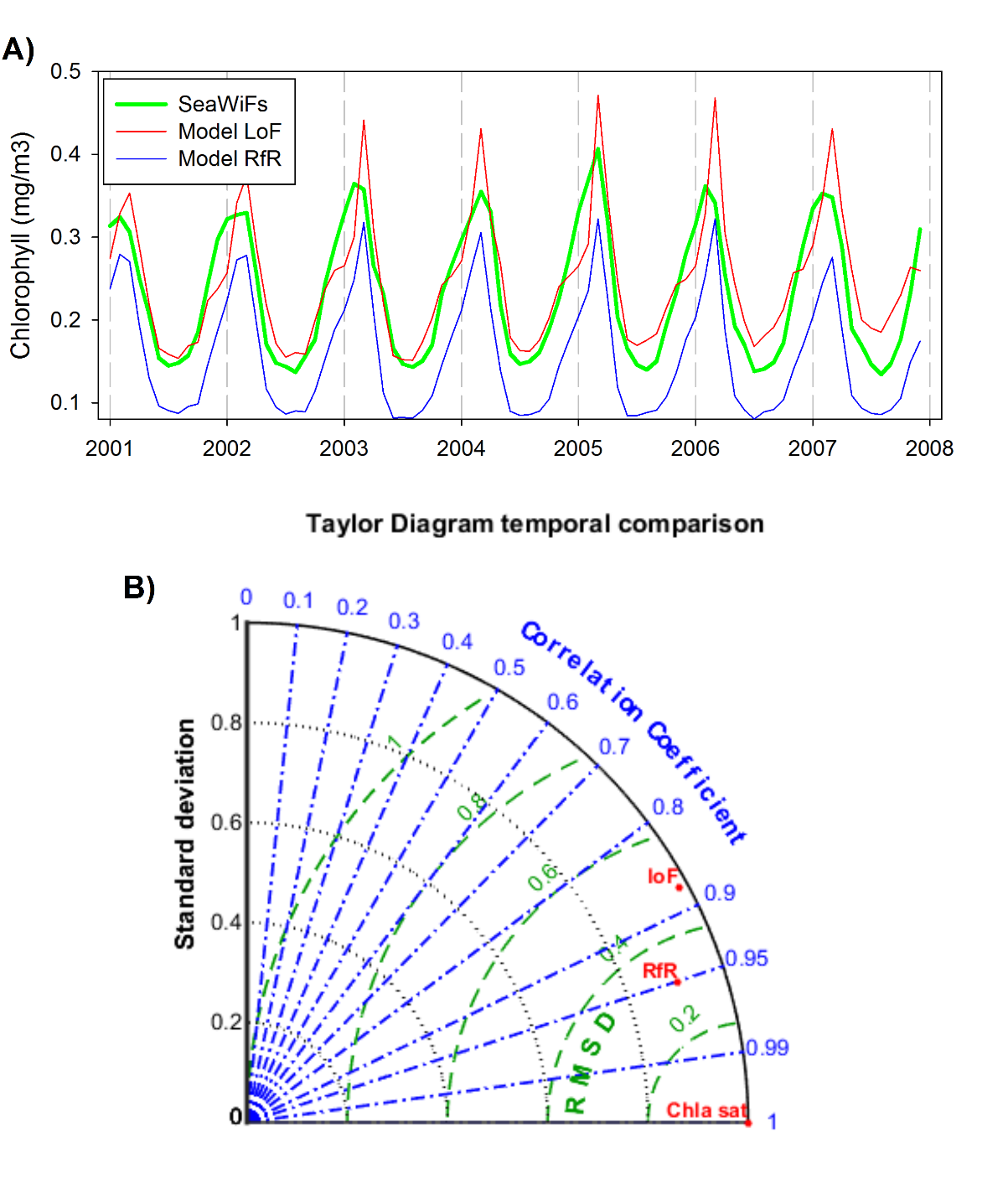


Figure S1. A) Temporal comparison of mean (basin wide) surface (0-10m) chlorophyll concentration from SeaWiFs (green line), RfR (blue line) and LoF (red line) simulations (mg/m3). B) Taylor Diagram of the chlorophyll time-series comparison shown in panel A.


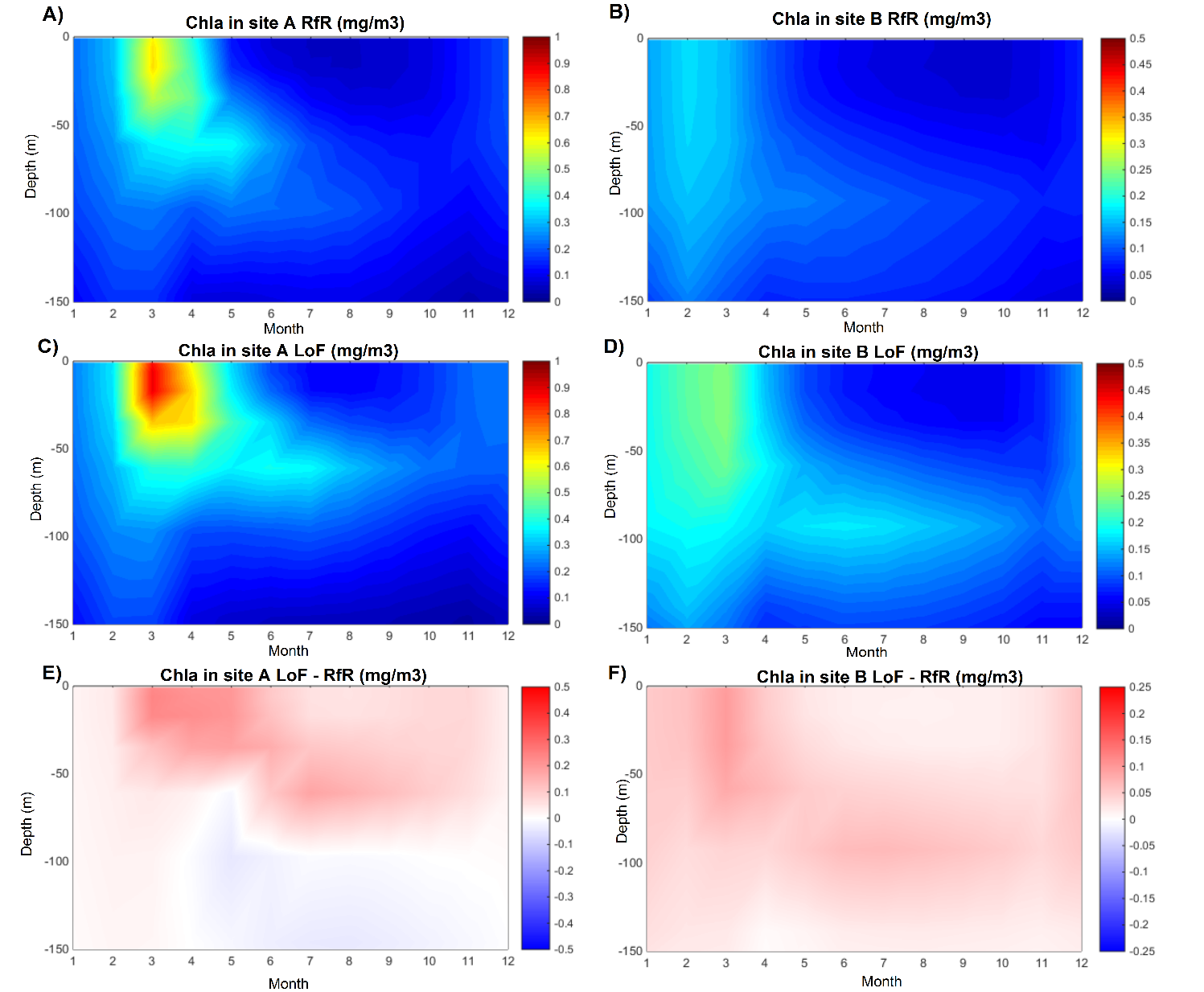


Figure S2. Vertical climatological chlorophyll distributions at the two sites (‘A’ & ‘B’) identified in Fig. 1 (mg/m3). A) Chlorophyll in site A for the RfR simulation (mg/m3). B) Chlorophyll in site B for the RfR simulation (mg/m3). C) Chlorophyll in site A for the LoF simulation (mg/m3). D) Chlorophyll in site B for the LoF simulation (mg/m3). E) Chlorophyll anomaly (RfR – LoF) at site A (mg/m3). F) Chlorophyll anomaly (RfR – LoF) at site B (mg/m3). Please note the different color scales for sites A and B.


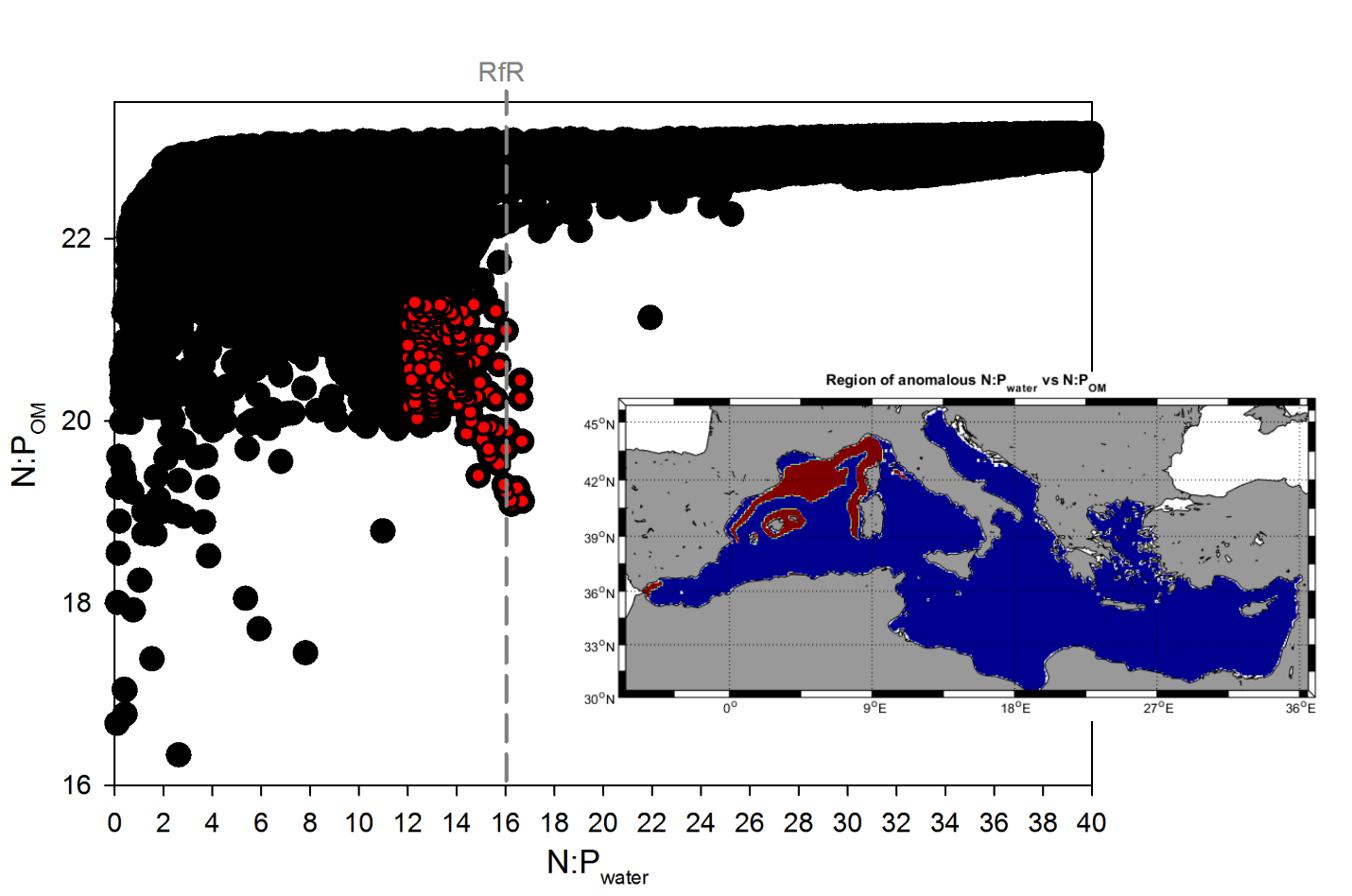


Figure S3. Scatter plot of N:P in OM versus N:P in water for the LoF run. The map in the inlet shows the region from where the red dots come from.
